# Supplementary material for: Patterns of Intron Gain and Loss in Fungi
Source: PLoS Biol. 2004 Nov 30;2(12):e422. doi: 10.1371/journal.pbio.0020422 (PMC532390; doi:10.1371/journal.pbio.0020422)
Supplement: Table S1 — Also available at http://genes.mit.edu/NielsenEtAl/. (4.3 MB ZIP). [file pbio.0020422.st001.zip › NielsenEtAl/html/678.html]

AN3894.1.NCU04280.1.MG08584.1.FG10198.1


```
 CLUSTAL W (1.82) Multiple Sequence Alignments - Introns Inserted


Sequence 1: MG08584.1	801 aa
Sequence 2: FG10198.1	811 aa
Sequence 3: NCU04280.1	809 aa
Sequence 4: AN3894.1	796 aa
Alignment Length: 818 aa
Number Identitical Residues: 465 aa
Alignment Score (without introns) 21502


MG08584.1 	MRPIGLSSRSGGILL0RIPR---AQAIRCCAG-------RRLYTTGPP-----LPPIYEK
NCU04280.1	MRSIDLASRSGAALK0RAIQAKQLPSRVACAS-------ARSFSSTSSNNTGVLPPRYET
FG10198.1 	MVSMEVASRSGGAMT0QLLRRSGPNAIRSTAALSSNKNSRRNFSSVKS-----LPPTYEK
AN3894.1  	MLRQIVSQRS-AARR0QLIDQLAPCLRRGLAT-------ATDSTPTSS-----RMPPYSK
          	*    ::.** .    :             *            :.  .       * *..

MG08584.1 	LYSNYSLVRKVLGRQR-LTLAEKILYSHLDDVEASLLNGTKNGTDIRGNANLQLKPDRVN
NCU04280.1	LYNKYSEVRRVLGSQR-LTLAEKILYSHLDNVEESLLSNTNGGRDVRGKANLKLKPDRVN
FG10198.1 	LYTKYTDVRRVLGKQR-LTLAEKILYSHLDNPEESLLSNTENGQNIRGKANLRLNPDRVN
AN3894.1  	IVQNLEQVRKVLGSSRALTLAEKILYSHLDNAEESLLTGTNNGRDIRGKADLKLKPDRVA
          	:  :   **:*** .*:*************: * ***..*:.* ::**:*:*:*:**** 

MG08584.1 	MQDASAQMALLQFMSCNLPQTAIPASIHCDHLIVGEKGAAEDLTSGINMNKEVFDFLESA
NCU04280.1	MQDASAQMALLQFMTCNLPQPVIPASIHCDHLIVGSKGADEDLKAGIATNNEVFDFLESA
FG10198.1 	MQDASAQMAILQFMSCNLAKPAIPASIHCDHLIVGSKGAKDDLSAGIETNKEVFDFLESA
AN3894.1  	MQDASAQMALLQFMSCGLPSTAVPASIHCDHMIVGERGADTDLPASIQGNKEVFDFLESA
          	*********:****:*.*....:********:***.:**  ** :.*  *:*********

MG08584.1 	ARKYGLDFWPPGAGIIHQTVLENYAIPGMMMLGTDSHTPNAGGLCTIAIGVGGADAVEAL
NCU04280.1	GKKYGIDFWPPGAGIIHQTVLENYAVPGLMMLGTDSHSPNAGGLTTITIGVGGADAVEAL
FG10198.1 	ARKYGMDFWPPGAGIIHQTVLENYALPGLMMLGTDSHSPNAGGLSTITIGVGGADAVEAL
AN3894.1  	SKRYGIEFWPPGAGIIHQSVLENYSAPGLMMLGTDSHTPNAGGLGAIAIGVGGADAVDAL
          	.::**::***********:*****: **:********:****** :*:*********:**

MG08584.1 	VGAPWELKAPKILNVHLTGKLSEWASPKDIILNLAGQLTVRGGTGYIIEYSGPGVDTLSA
NCU04280.1	VGAPWELKAPKILGVRLTGKLNDWVSAKDIILHLTGRLTVRGGTGYIVEYFGPGIESLSC
FG10198.1 	VGAPWELKAPKVLGVHLVGKLSNWASPKDVILRLAGHLTVRGGTGSIIEYFGEGVDSLSA
AN3894.1  	VDAPWELKAPRILGVRLEGKLQGWAAPKDIILHLAGKLTVRGGTGFVIEYHGPGVETLST
          	*.********::*.*:* ***. *.:.**:**.*:*:******** ::** * *:::** 

MG08584.1 	TGMATVTNMG~AEVGATTSIFPYTPASARYLKSTRREAAANAASMLQTYPGVGAGDDQYF
NCU04280.1	SGSSTLCNMG2AEIGATTSIFPFTEASSRYLKATNREQADKDAKNFENFT-KGTGQDSFF
FG10198.1 	TGMATICNMG2AEVGATTSIFPYTKASARYLDSTRRSQANKNIEALESFAGNSSDPDARW
AN3894.1  	TGMATICNMG~AEVGATTSLFPFSPNHVPYLKATNRADVAEAAAKIASAG-------SSS
          	:* :*: *** **:*****:**::     **.:*.*  . :    : .            

MG08584.1 	RFEADKGAEFDQQVNIDLSTLEPHINGPFTPDLSTPLSKFKETIKAEGWPETLSAGLIGS
NCU04280.1	KFKADEGAQYDELIEIDLSKLEPHINGPFTPDLSIPLSEFKKTVQEQKWPEKLSAGLIGS
FG10198.1 	QFKADEGAEYDDLITIDLSTLEPHINGPFTPDLATPLSKFKEVVKEQEWPELLSAGLIGS
AN3894.1  	LLRADTSAEYDELITIDLSTLEPHINGPFTPDLSVPLSRFAETVRKNNWPETFNAGLIGS
          	 :.** .*::*: : ****.*************: ***.* :.:: : *** :.******

MG08584.1 	CTNSSYEDMTRVESMLKAAGEAGLKPAADFYITPGSEQIRSTLERDGTLETFTDAGGIVL
NCU04280.1	CTNSSYEDMTRCESIVKAAEQAGIKPASDFYITPGSEGIRATLERDNTLQTFEKAGGIVL
FG10198.1 	CTNSSYEDMTRVESLLKDAQKAGLKPAADFYITPGSEQIRATLERDGTLQTFQQAGGIVL
AN3894.1  	CTNSSYEDMTRAEHLVKQANAAGLKPKADLFITPGSEQIRATLDRDQTLSTFSSAGGTVL
          	*********** * ::* *  **:** :*::****** **:**:** **.** .*** **

MG08584.1 	SNACGPCIGQWKRQDNIPKGQPNAILTSYNRNFRGRNDGNPGTMNFLASPEIVTAMAFAG
NCU04280.1	SNACGPCIGQWKRQDDIPKGTPNAILTSYNRNFRGRNDGNLETMNFLASPEIVTAMAYAG
FG10198.1 	SNACGPCIGQWKRQDGVEKGTSNAILTSYNRNFRGRNDGNPDTMNFLASPEIVTAMAFAG
AN3894.1  	ANACGPCIGQWKRTDDVPKGTDNAIFTSYNRNFPGRNDGNRRTMNFLASPELVTALTYAG
          	:************ *.: **  ***:******* ******  *********:***:::**

MG08584.1 	STTFNPVTDSLKTPDGKDFKFPPPQGLEGPAAPFDSGVEELLPLCQQPNPQVDVKISPTS
NCU04280.1	STTFNPITDSLTTPDGKEFKFPAPKGLEGPETPFEAGREVFQVQSGSPNPNVEVAISPSS
FG10198.1 	STTFNPVTDSIKTPDGKDFKFSPPHGLEGPQTPFESGNAELGVLSQEPDPNTKIAISPTS
AN3894.1  	STTFNPVTDSITTPSGSEFRFEPPTGQDLPSKGFEAGNPAFQPSAPVPDSSVEVKVSPTS
          	******:***:.**.*.:*:* .* * : *   *::*   :   .  *:....: :**:*

MG08584.1 	ERLALLEPFDSFPEHDLKGLRVLVKVTGKCTTDTISAAGPWLKYKGHLPNISTNTLNTAI
NCU04280.1	ERLALLEPFEPFPESDLSGLRVLVKVTGKCTTDTISAAGPWLKYKGHLPNISENTLNTAV
FG10198.1 	ERLAFLDPFPAFPSSNLSGLRVLVKVTGKCTTDTISAAGPWLKYKGHLPNISTNTLNTAI
AN3894.1  	TRLALLEPFAPFPNSDLQNLSVLYKVKGQCTTDTISAAGPWLKYKGHLPNISANTLIGAV
          	 ***:*:** .**. :*..* ** **.*:*********************** ***  *:

MG08584.1 	NAATGEVNAAYDLDGSKHTIPDLAARWRAAGREWVVVAEHNYGEGSAREHAALQPRYLGC
NCU04280.1	NAATGEVNVAYDLDGSKHTIPELAKQWKQRNQPWLVVAEHNYGEGSAREHAALQPRYLGA
FG10198.1 	NKETGEVNAAYDLDGSKHTIPELGQLWKDRGQEWLVVAEHNYGEGSAREHAALQPRYLGA
AN3894.1  	NAATGETNVAYDEAGKQHTIPDLAAQWKAQGREWLVVAEENYGEGSAREHAALQPRYLGG
          	*  ***.*.***  *.:****:*.  *:  .: *:****.******************* 

MG08584.1 	RAIVTKSFARIHETNLKKQGVVPLTFAEEIDYDKIEAGDEVETVGLYDMLKNGGQGEVQL
NCU04280.1	RIIVTKSFARIHETNLKKQGVVPLTFANEQDYDLIDAGDEVATVGLYEMLKNGGQGEVSL
FG10198.1 	RVVLTKSFARIHETNLKKQGVVPLTFEDEADYDKIAAGDEVTTVGLYEMLQNGGKGDVQL
AN3894.1  	RVILAKSFARIHETNLKKQGVVPLTFADKADYDRIDACDVVATEGLYETLKNGGKGEVKL
          	* :::********************* :: *** * * * * * ***: *:***:*:*.*

MG08584.1 	RVTKGD-GSEVMIPVKHAVSKDQAGFILAGSALNMLSKRN
NCU04280.1	RVTKKDTGKVVEIKTKHAVSKDQAGFILAGSALNLLAKGN
FG10198.1 	RVTKSS-GEEVLIPTKHAVTKDQAGFILAGSALNLLSKGI
AN3894.1  	RVTKKS-GEEIVIPVKHTLSADQSSFILAGSALNVLSKR-
          	**** . *. : * .**::: **:.*********:*:*
```
